# Phase II study of zevorcabtagene autoleucel, a fully human BCMA-targeting CAR T cell therapy, in patients with relapsed/refractory multiple myeloma

**DOI:** 10.1186/s40164-025-00710-y

**Published:** 2025-09-30

**Authors:** Wenming Chen, Chengcheng Fu, Baijun Fang, Aibin Liang, Zhongjun Xia, Yanjuan He, Jin Lu, Hui Liu, Ming Hou, Zhen Cai, Wei Yang, Siguo Hao, Songfu Jiang, Hongmei Jing, Jing Liu, Xin Du, Rong Fu, Heng Mei, Zunmin Zhu, Yanli Yang, Hong Liu, Xingxing Meng, Nishanthan Rajakumaraswamy, Daijing Yuan, Huamao Wang, Zonghai Li

**Affiliations:** 1 Department of Hematology, Beijing Chao-Yang Hospital, Capital Medical University, Beijing, 100020 China; 2 The First Affiliated Hospital of Soochow University, Suzhou, 215006 China; 3 Henan Cancer Hospital, Zhengzhou, China; 4 Tongji Hospital of Tongji University, Shanghai, China; 5 Department of Hematology, Sun Yat-sen University Cancer Center, Guangzhou, China; 6 Department of Hematology, Xiangya Hospital, Central South University, Changsha, China; 7 Peking University People’s Hospital, Beijing, China; 8 Department of Hematology, Beijing Hospital, Beijing, China; 9 Department of Hematology, Qilu Hospital of Shandong University, Jinan, China; 10 The First Affiliated Hospital, Zhejiang University School of Medicine, Hangzhou, China; 11 Shengjing Hospital of China Medical University, Shenyang, China; 12 Xinhua Hospital Affiliated to Shanghai Jiaotong University School of Medicine, Shanghai, China; 13 The First Affiliated Hospital of Wenzhou Medical University, Wenzhou, China; 14 Department of Hematology, Peking University Third Hospital, Beijing, China; 15 Department of Hematology, The Third Xiangya Hospital of Central South University, Changsha, China; 16 Division of Hematology, The Second People’s Hospital of Shenzhen, The First Affiliated Hospital of Shenzhen University, Shenzhen, China; 17 Department of Hematology, Tianjin Medical University General Hospital, Tianjin, China; 18 Union Hospital, Tongji Medical College, Huazhong University of Science and Technology, Wuhan, China; 19 Department of Hematology, Henan Provincial People’s Hospital, Zhengzhou, China; 20 The First Affiliated Hospital of Bengbu Medical College, Bengbu, China; 21 Affiliated Hospital of Nantong University, Nantong, China; 22 CARsgen Therapeutics Co. Ltd, Shanghai, China

**Keywords:** BCMA, CAR T cells, Chimeric antigen receptor T cell, Zevor-cel, Zevorcabtagene autoleucel, CT053, Relapsed/refractory multiple myeloma, Targeted immunotherapy

## Abstract

**Background:**

Zevorcabtagene autoleucel (zevor-cel) is a fully human autologous CAR T-cell therapy targeting B-cell maturation antigen approved in China since 2024 for patients with relapsed/refractory multiple myeloma (RRMM).

**Methods:**

LUMMICAR STUDY 1 is a phase 2, single-arm study conducted across 23 centers in China. RRMM patients aged ≥ 18 to ≤ 75 years with measurable disease who had received ≥ 3 prior lines of therapy, with adequate organ function and bone marrow reserve, with an Eastern Cooperative Oncology Group (ECOG) score of 0–1, were eligible. Patients previously treated with any CAR T-cell therapy, or any BCMA-directed therapy were ineligible. The primary endpoint was objective response rate (ORR) determined by an Independent Review Committee. The secondary endpoints included ORR determined by investigator, additional efficacy outcomes including complete response (CR)/ stringent complete response (sCR) rate, duration of response (DOR), minimal residual disease negativity, safety outcomes including incidence and severity of adverse events, and pharmacokinetics of zevor-cel.

**Results:**

Overall, 125 patients underwent apheresis, 105 patients received lymphodepletion, 102 patients (median age of 59.5 [range: 38, 75] years; 53.9% male and 46.1% female) received zevor-cel. The ORR was 92.2% (95% CI 85.13–96.55) with 70 patients (68.6%) achieving sCR and 3 (2.9%) achieving CR. At a median follow-up of 20.3 (interquartile range [IQR] 12.5, 23.8) months, 45 (44.1%) progression-free survival (PFS) events and 20 (19.6%) overall survival (OS) events were observed, the DOR, PFS and OS data were not mature. Cytokine release syndrome was reported in 92 (90.2%) patients, with grade 3 or 4 events in 7 (6.9%) patients. Immune effector cell associated neurotoxicity syndrome was reported in 2 patients at grade 1; no zevor-cel-related grade ≥ 3 neurotoxicity occurred.

**Conclusion:**

Zevor-cel induces deep and durable responses in heavily pre-treated RRMM patients with a manageable safety profile.

**Supplementary Information:**

The online version contains supplementary material available at 10.1186/s40164-025-00710-y.

## Introduction

Multiple myeloma (MM) accounts for 10% to 15% of all hematologic malignancies [1]. The 5-year survival rate of myeloma is 57.9% in the United States [2]. In China, over the last three decades, the burden of MM has doubled, with significant increase in incidence [3]. Chinese patients with MM have inferior prognosis due to delayed diagnosis and limited accessibility to novel drugs [4]. The 5-year OS rate was lower than 50% in the three most popular academic multiple myeloma centers, with only half of the patients receiving bortezomib-based regimens [5]; These findings corroborate the results of a retrospective single-center study in China that showed a 5-year OS of 47.6% [6].

Despite the unprecedented therapeutic advances in MM over the last two decades, majority of the patients still relapse and develop refractory disease [7–10]. Recently, chimeric antigen receptor (CAR) T-cell therapies were added to the therapeutic armamentarium of physicians treating MM [6]. The B-cell maturation antigen (BCMA)-targeting CAR T-cell therapies, ciltacabtagene autoleucel (cilta-cel) [11] and idecabtagene vicleucel (ide-cel), have been approved by the US Food and Drug Administration (FDA) [12] for RRMM. These two CAR T-therapies have achieved remarkable overall response rates of 73%–100% and complete response (CR) or stringent CR (sCR) rates ranging from 33%–83% [13–17]. China’s large population requires highly efficacious and affordable treatment options but the accessibility to commercially available CAR T products in China has been limited. Equecabtagene autoleucel (eque-cel), a BCMA-targeting CAR-T cell therapy manufactured in China, has been approved by China’s National Medicinal Products Administration (NMPA) for patients with RRMM who have undergone at least 3 prior lines of treatment. In a study of 103 patients, eque-cel demonstrated a 12-month PFS rate of 78.8% and a response rate of 96.1%. Despite the compelling efficacy observed with CAR-T therapies, safety issues remain a paramount concern including cytokine release syndrome (CRS), neurotoxicity, especially parkinsonism, infections along with the development of second primary malignancies. Non-relapse mortality was reported in 6% of the patients treated with cilta-cel, mainly due to infections, and in 5% of patients treated with ide-cel [16, 18]. 

Zevorcabtagene autoleucel (zevor-cel or CT053) is a BCMA-directed CAR T-cell therapy consisting of fully human anti-BCMA single chain variable fragment (scFv), CD8α hinge/spacer domain, CD8α transmembrane domain, 4-1BB intracellular domain and CD3ζ cytoplasmic signalling domain that are linked sequentially [19]. Zevor-cel employs a third-generation self-inactivated lentivirus system, offering enhanced safety compared to the first- and second-generation vectors used in CAR T cell preparation [20]. In an investigator-initiated exploratory study, zevor-cel demonstrated acceptable safety profile with 100% ORR [21]. Subsequently, in the phase 1 of LUMMICAR STUDY 1 (NCT03975907), zevor-cel induced durable responses with an ORR of 100% with a tolerable safety profile [22]. Zevor-cel was approved in February 2024 by NMPA in China [22, 23]. Herein, we present the results from the pivotal phase 2 cohort of LUMMICAR STUDY 1 that led to the approval of zevor-cel in China.

## Methods

### Study design and patients

**Fig. 1:**
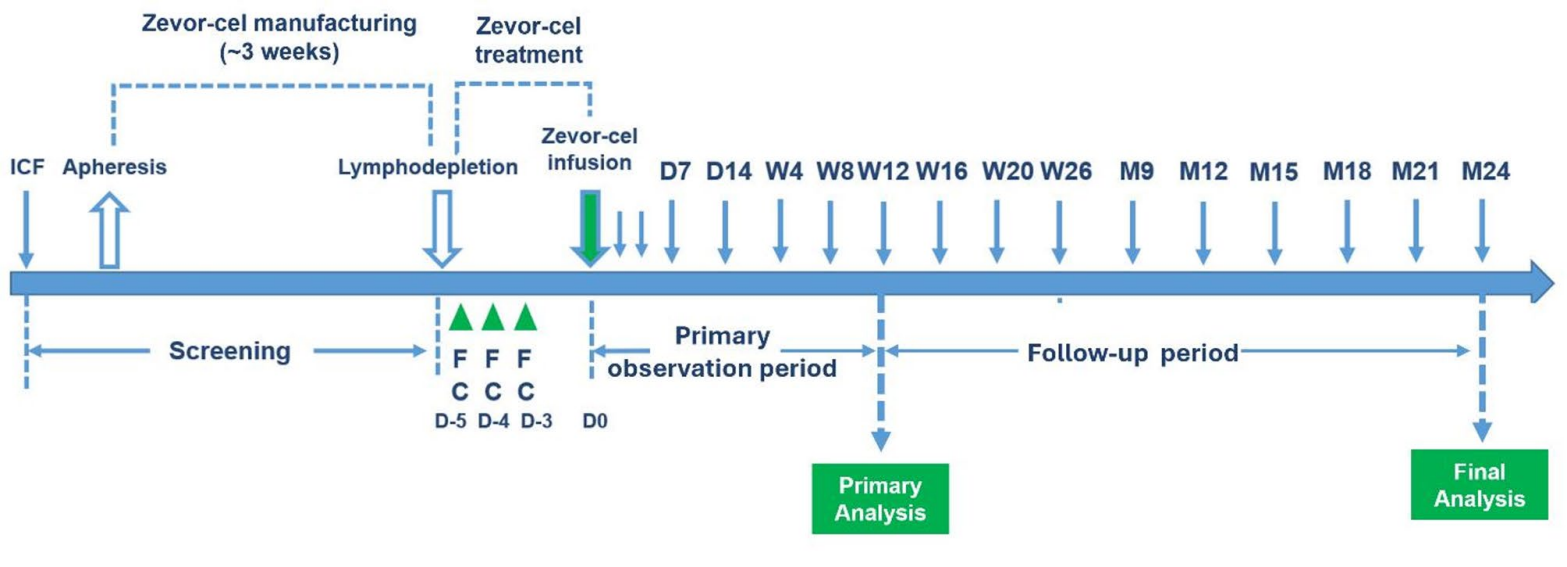
Study design. *C* cyclophosphamide (300mg/m^2^); *D* day; *F* fludarabine (25 mg/m^2^); *ICF* informed consent form; *M* month; *W* week

LUMMICAR STUDY 1 (NCT03975907) is a pivotal, single-arm, open-label, multi-center, phase 2 study assessing the efficacy, safety, and pharmacokinetics (PK) of zevor-cel in patients with RRMM conducted at 23 centers across China. Zevor-cel was manufactured from autologous peripheral blood mononuclear cells (PBMCs), which were collected and isolated at the study center and then transported to CARsgen Therapeutics manufacturing site for further processing. If clinically necessary, bridging therapy was permitted after apheresis consisting primarily of agents and combinations that the patients had previously been exposed to. Patients were lymphodepleted with fludarabine at 25 mg/m^2^ and cyclophosphamide at 300 mg/ m^2^ daily for 3 consecutive days. Zevor-cel was administered as a single infusion at a target dose of 150 × 10^6^ or 180 × 10^6^ (for patients with body weight > 80 kg at the discretion of investigator) BCMA CAR- positive T cells 1 to 2 days after the completion of lymphodepletion. Patients were followed up for 24 months for efficacy and safety and then entered the long-term follow-up period for up to 15 years (Fig. 1). Prophylaxis against infusion-related reactions was administered up to 30 min prior to infusion consisting of anti-histamines (promethazine 12.5–50 mg or equivalent) and antipyretics (indomethacin suppositories 50–100 mg or equivalent).

All patients were managed per standard institutional and/or national guidelines for prophylactic and empirical antimicrobial therapy. Patients with evidence of prior hepatitis B infection (positive for HBcAb), received prophylactic antiviral therapy starting prior to lymphodepletion with close monitoring of hepatic function. Immunoglobulin replacement therapy (IGRT) was administered to patients with persistent hypogammaglobulinemia (IgG ≤ 400 mg/dL) and to those with serum IgG 400–600 mg/dL experiencing severe, persistent, repeated infections, or co-existing neutropenia or grade ≥ 3 CRS.

### Key inclusion criteria

RRMM patients aged ≥ 18 to ≤ 75 years, previously treated with at least 3 lines of therapy including at least an immunomodulatory drug and a proteasome inhibitor, having relapsed on or deemed refractory to the last line of therapy, with an Eastern Cooperative Oncology Group (ECOG) score of 0–1 were enrolled in the study. All had measurable disease defined by the presence of one of the following: (1) Serum M-protein ≥ 10 g/L, (2) Urine M-protein ≥ 200 mg/24 hours, or (3) or abnormal serum free light chain (FLC) ratio with involved FLC ≥ 100 mg/L if both serum and urine M protein were not measurable at baseline.

Adequate bone marrow reserve was required as indicated by the following (without requiring supportive therapy or ≥ 2 transfusions within 7 days of testing): ANC ≥ 1.0 × 10^9^/L, platelet count ≥ 75 × 10^9^/L (if the proportion of plasma cells in the bone marrow is > 50%, platelets ≥ 50 × 10^9^/L), Hb ≥ 70 g/L, absolute lymphocyte count (ALC) ≥ 0.4 × 10^9^/L. In addition, creatinine clearance ≥ 40 mL/min, alanine aminotransferase (ALT) ≤ 2.5 × upper limit normal (ULN), aspartate aminotransferase (AST) ≤ 2.5 × ULN, total bilirubin ≤ 2 × ULN were required.

### Key exclusion criteria

Patients previously treated with any CAR T-cell therapy, any BCMA-directed therapy, previous allogeneic stem-cell transplantation at any time or autologous stem cell transplantation within 12 weeks of leukapheresis were ineligible. Patients with significant organ dysfunction such as uncontrolled congestive heart failure, angina, myocardial infarction, stroke (except lacunar infarct), coronary artery bypass graft, clinically significant cardiac arrhythmias, uncontrolled diabetes, pulmonary embolism, left ventricular ejection fraction (LVEF) < 50% before leukapheresis were excluded. A complete description of the study design and full inclusion and exclusion criteria can be accessed at http://www.chinadrugtrials.org.cn/clinicaltrials.searchlist.dhtml. The study was conducted in accordance with the principles of the Declaration of Helsinki and International Council on Harmonisation guidelines for Good Clinical Practice. Written informed consent was obtained from all patients prior to study entry.

### Endpoints and assessments

The primary endpoint was ORR per the International Myeloma Working Group (IMWG) 2016 criteria as adjudicated by an Independent Review Committee (IRC). The secondary endpoints included ORR by investigator, additional efficacy outcomes including the rate of CR/sCR, rate of very good partial response or better (≥ VGPR), duration of response (DOR), progression-free survival (PFS), overall survival (OS), time to response (TTR), minimal residual disease (MRD) negativity rate, safety outcomes including incidence and severity of treatment-related AEs, AEs of special interest (including CRS and neurotoxicities), laboratory abnormalities, immunogenicity and pharmacokinetics (PK) of zevor-cel.

Laboratory assessments for efficacy including serum protein electrophoresis (SPEP), serum immunofixation electrophoresis (SIFE), urine protein electrophoresis (UPEP from 24-hour urinary protein), urine immunofixation electrophoresis (UIFE), serum free light chain (FLC) and its ratio, bone marrow immunohistochemistry, and bone marrow MRD were analyzed at a central laboratory.

Response to zevor-cel was assessed on W4, W8, W12, W16, W20, W26, M9, M12, M15, M18, M21 and M24 post-infusion per IMWG 2016. Thereafter, follow-up was conducted every 6 months until disease progression, death, withdrawal of consent, or completion of long term follow up.

Adverse events (AEs) were graded per the National Cancer Institute (NCI) common terminology criteria for AEs (CTCAE) version 5.0. Cytokine release syndrome (CRS) and immune effector cell-associated neurotoxicity syndrome (ICANS) were graded per the American Society for Transplantation and Cellular Therapy (ASTCT) 2019 criteria [24]. 

MRD was evaluated using EuroFlow at a sensitivity threshold of 10^− 5^. In vivo expansion and persistence of zevor-cel were assessed by quantitative polymerase chain reaction (qPCR) and was measured using copies per microgram of genomic DNA. The start date of CRS was defined as the date of pyrexia or any other symptoms of CRS as determined by the investigator, which could not be explained by alternative diagnoses. The ICE score was recorded before lymphodepletion (used as the reference for the evaluation of immune effector cell-associated neurotoxicity syndrome [ICANS]) then at D3, D7 and on discharge from inpatient stay. If there were new neurological or psychiatric symptoms or signs, ICE score was recorded and followed up until resolution or baseline level was reached.

### Statistical analysis

Based on the collective results for the ORR (approximately 30%) of the previously approved non-CAR T agents for the treatment of RRMM, the null hypothesis ORR achieved by zevor-cel at week 12 was set as 50%. Based on the preliminary data from the exploratory clinical trials of zevor-cel, it was assumed that the ORR at week 12 after zevor-cel infusion was 75%. To detect an ORR difference of 25% with 90% power, 42 patients would be needed. In order to adequately characterize the safety profile of zevor-cel, at least 100 patients were enrolled.

The modified intention-to-treat set and the safety analysis set, both were defined as all patients who received a zevor-cel infusion, were analysed independently for efficacy and safety. MRD results were summarized using the number and percentage of patients at specified timepoints after zevor-cel infusion, along with two-sided Clopper-Pearson 95% CIs. Time-to-event efficacy endpoints such as PFS, DOR, and OS were estimated using the Kaplan-Meier method, and 95% CIs were estimated using the Brookmeyer-Crowley method. Safety analyses were performed to assess the incidence of treatment-emergent adverse events (TEAEs). PK analyses were performed using descriptive statistics. All statistical analyses and plots were conducted using SAS software, version 9.4 or higher.

## Results

### Baseline and disease characteristics

Between 01 December 2020 and 15 January 2022, a total of 125 patients were enrolled and underwent apheresis from 23 centers. A total of 20 patients discontinued after apheresis, including four patients who withdrew consent, 15 patients who did not meet the criteria for lymphodepletion and one patient who experienced rapid disease progression. Overall, 105 patients underwent lymphodepletion of whom, 3 patients did not proceed to receive zevor-cel infusion, 2 due to rapid disease progression and 1 patient due to not meeting the eligibility criteria for zevor-cel infusion. A total of 102 patients received zevor-cel infusion. The median time from apheresis to zevor-cel infusion was 33.0 (range: 20–99) days. Twenty-six (25.5%) received bridging therapy.

**Fig. 2:**
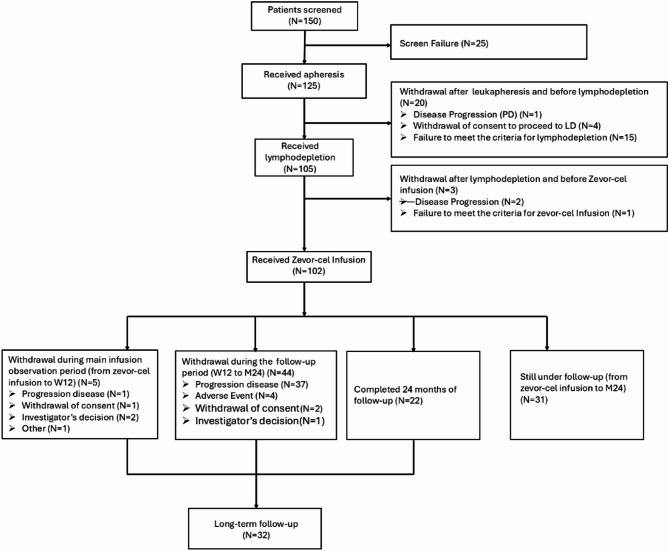
Patient disposition. The patient flow chart through the study from screening to long-term follow-up. *BCMA* B-cell maturation antigen; *CAR-T* chimeric antigen receptor T-cell; *N* number of patients

As of data cut-off date, 22 patients had completed the initial 24-month follow-up period, 31 patients were still under the initial 24-month follow-up period, and 49 patients had withdrawn from the initial 24-month follow-up period (5 patients during the first 12 weeks and 44 patients between W12 and M24). Of all patients who received zevor-cel infusion, 32 patients had entered the long-term follow-up period, which include patients who completed 24-month follow-up period (*n* = 22) and patients who withdrawn before 24-month follow-up (from any study-related assessments) but did not withdraw their consent for survival follow-up (Fig. 2).

The median age of the infused patient population (*n* = 102) was 59.5 (IQR 53.0, 66.0) years, 55 of 102 patients (53.9%) were male, and all were Chinese by ethnicity (Table 1). A total of 39 patients (38.2%) presented with the International Staging System (ISS) stage III disease, while 46 (45.1%) patients had at least one high-risk cytogenetic abnormality. Extramedullary disease as assessed by the IRC was present in 11 (10.8%) patients.

The median number of prior lines of therapy was 4.0 (range: 3, 15) and 24 (23.5%) had received prior hematopoietic stem-cell transplantation. Overall, 91 (89.2%) patients were double-class refractory, 23 (22.5%) were triple-class refractory, and 28 (27.5%) were refractory to an anti-CD38 agent.

### Efficacy of zevor-cel

#### Response rates

**Fig. 3:**
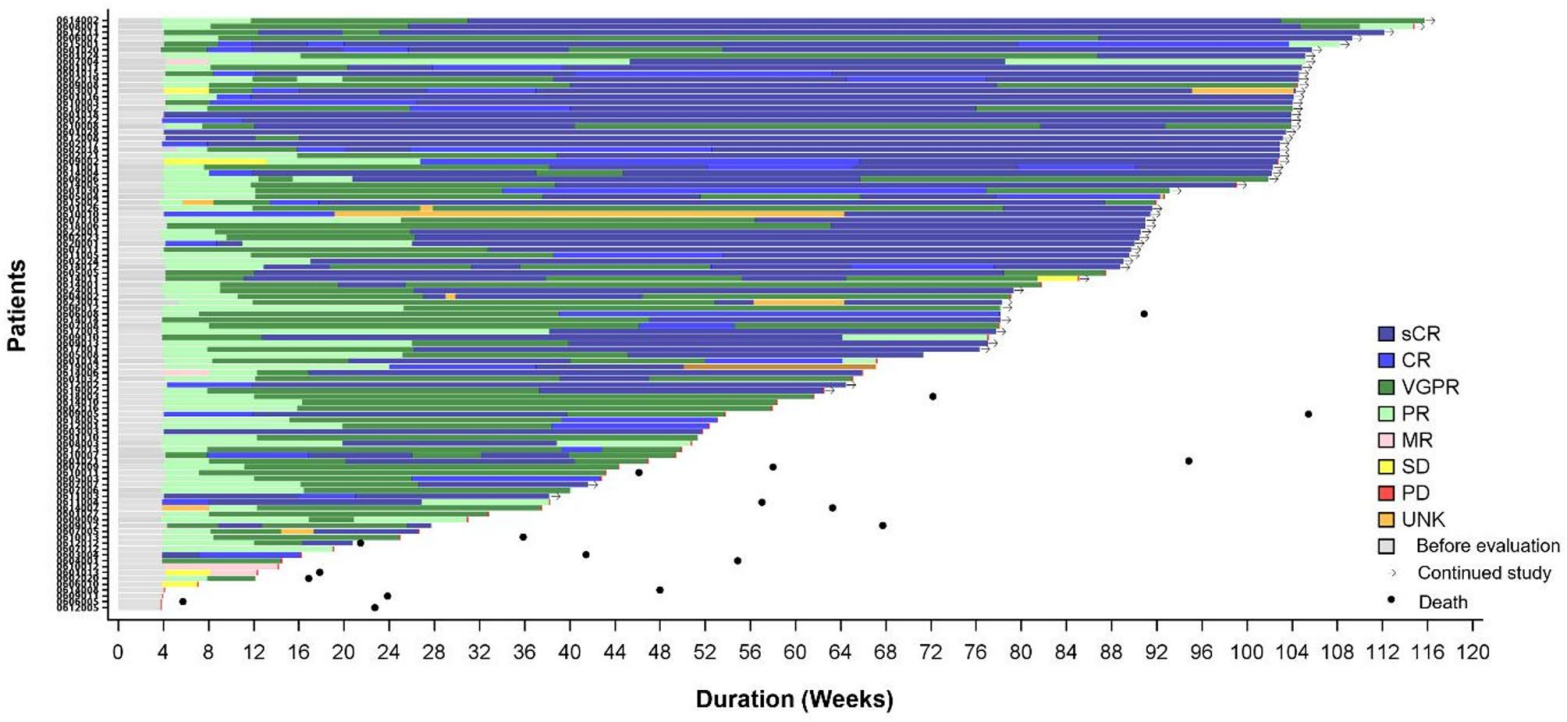
Swimmer plot of outcomes post zevor-cel infusion. The y-axis represents individual patient numbers of the 102 patients with responses. The x-axis is time in days since zevor-cel infusion. Responses were evaluated by IRC. The length of the bars corresponds to the time each patient spent in the study. The tumour assessment results include the overall efficacy assessment at each time point, and patients without arrows indicate that they have withdrawn from the group. *CR* complete response; *IRC* independent review committee; *MR* minimal residual disease; *PD* progressive disease; *PR* partial response; *sCR* stringent CR; *SD* stable disease; *UNK* unevaluable; *VGPR* very good partial response

At the time of data cut-off (17.9 months after the last patient received zevor-cel), the median duration follow-up was 20.3 (IQR 12.5, 23.8) months for all infused patients, and 24.7 (range: 23.3, 31.4) months for those (*n* = 32) who had entered the long-term follow-up. Of the 102 evaluable patients, the objective response rate (ORR) was 92.2% (94 responders; 95% CI 85.1%–96.6%), with 70 (68.6%) patients achieving sCR, 3 (2.9%) patients achieving CR, 20 (19.6%) patients achieving VGPR, and 1 patient (1%) achieving PR (Table 2). The VGPR or higher response rate was 91.2% (93/102), CR/sCR rate was 71.6% (73/102). A trend towards deepening of response was noted with longer duration of follow-up; the median TTR and the median time to reach CR/sCR, were 29.0 days (range: 26, 93) and 146 days (range: 28, 609), respectively. Out of 99 patients with MRD evaluable samples, 91.9% (91/99; 95% CI, 84.7%–96.5%) achieved MRD negativity with a threshold of 10^− 5^. The MRD negativity rate amongst patients achieving CR/ sCR and those with VGPR or better response were 100% (73/73, 95% CI 95.1%–100%) and 95.7% (88/92, 95% CI 89.2%–98.8%), respectively. The TTR of individual patients as well as other details including time to CR, VGPR, stable disease (SD), PD are shown in the swimmer plot (Fig. 3).

#### Duration of response, progression-free survival, overall survival

**Fig. 4:**
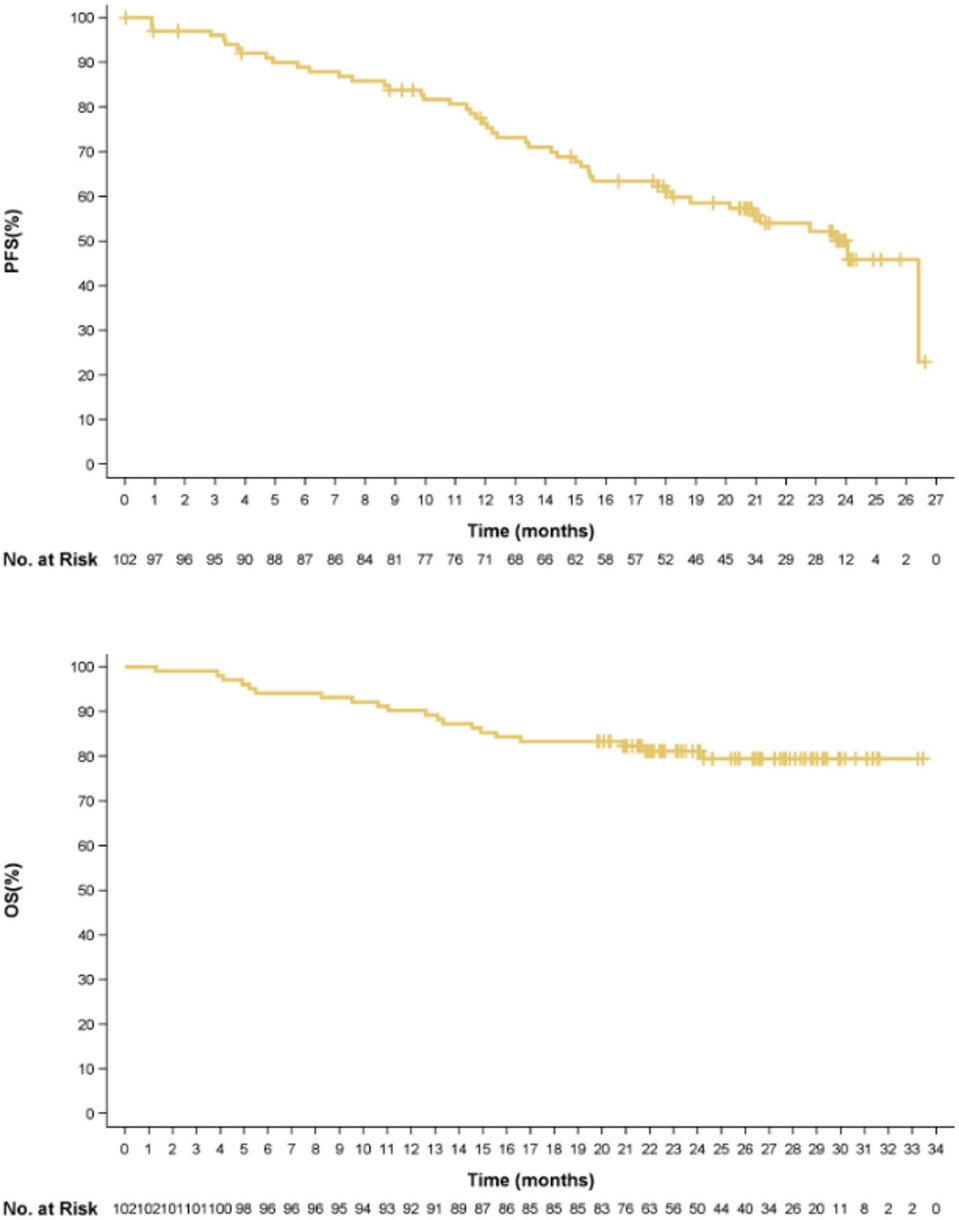
Kaplan Meier plots for PFS and OS. *PFS* progression free survival; *OS* overall survival

With the median follow-up of 20.3 (IQR 12.5, 23.8) months, 45 (44.1%) PFS events and 20 (19.6%) OS events were observed in the infused population (*n* = 102), Kaplan Meier plot for PFS and OS are shown in Fig. 4. The 12- and 18-month PFS rates were 76.3% (95% CI: 66.5%–83.6%) and 61.9% (95% CI: 51.2%–70.9%) respectively. A total of 41 (40.2%) patients reported disease progression. The estimated percentage of patients with response duration ≥ 12 months, ≥ 18 months were 76.8% (95% CI: 66.7%–84.2%) and 62.4% (95% CI: 51.2%–71.6%) respectively. The OS rates at 12, 18 and 30 months were 90.2% (82.6%–94.6%), 83.3% (74.6%–89.3%), and 79.4% (69.7%–86.3%) respectively. The overall survival (OS), DOR and PFS data were not mature by the data cut-off date.

In 73 (71.6%) patients with confirmed CR/sCR, the PFS event free rate and survival rate was higher and response duration were also longer: the PFS event free rates at 12 and 18 months were 88.8% (78.8%–94.2%) and 78.6% (67.0%–86.5%) and the estimated percentage of patients with a DOR ≥ 12, and ≥ 18 months were 85.9% (95% CI: 75.4%–92.2%) and 76.9% (95% CI: 65.0%–85.2%) respectively. The OS rates at 12, 18 and 30 months were 95.9% (87.8%–98.7%), 93.2% (84.3%–97.1%), and 87.7% (76.6%–93.8%), respectively.

#### Safety of zevor-cel

TEAEs and zevor-cel-related AEs were reported in all patients. The most common zevor-cel-related TEAEs were cytopenias, with at least 1 grade ≥ 3 haematological toxicity in all patients, including 90 (88.2%) patients with grade 4, and none with grade 5 (Table 3). Majority of grade ≥ 3 haematological toxicities resolved within 30 days post infusion. Based on laboratory data obtained within 30 days post infusion, 95 (93.1%) patients reported grade 3/4 neutropenia, of whom 87 (91.6%) recovered within 30 days post infusion; 63 (61.8%) patients reported grade 3/4 thrombocytopenia, of whom 46 (73.0%) recovered within 30 days post infusion; 100 (98.0%) patients reported grade 3/4 lymphopenia, of whom 99 (99.0%) recovered within 30 days post infusion; 61 (59.8%) patients reported grade 3/4 anaemia, of whom 52 (85.2%) recovered within 30 days post infusion.

CRS of any grade was reported in 92 (90.2%) patients, with the majority classified as grade 1 (*n* = 52, 51.0%) and grade 2 (*n* = 33, 32.4%); 5 patients (4.9%) experienced grade 3 and 2 (2.0%) suffered grade 4 events (Table 4). The median time to first onset of CRS was 4 days (range: 1, 14) days, and the median duration was 6 days (range: 1, 22) days. All CRS had resolved at the time of data cut-off, with 66 (66/92) patients receiving treatment with tocilizumab, 25 (25/92) receiving corticosteroids. ICANS was reported in only 2 patients (2.0%) both at grade 1; both patients made a full recovery without the need for tocilizumab or corticosteroids. Zevor-cel-related nervous system disorders were reported in 19 (18.6%) patients in our study. The most common zevor-cel-related nervous system disorders were dizziness reported in 8 (7.8%) patients and headaches reported in 8 (7.8%) patients, both of which were at grade 1 or 2. There were no movement disorders, neurocognitive deficits, parkinsonism, cranial nerve palsies, or Guillain-Barre syndrome reported. There were no zevor-cel related grade 3 or higher neurological AEs reported. Zevor-cel-related infections and infestations were reported in 54 (52.9%) patients, the grade ≥ 3 infections were reported in 28 (27.5%) patients, including 1 (1.0%) grade 5 pneumonia. Hypogammaglobulinemia was reported in 27 (26.5%) patients, including 5 (4.9%) grade ≥ 3 hypogammaglobulinemia.

A total of 52 (51.0%) patients reported SAEs, with 37 (36.3%) patients experiencing SAEs related to zevor-cel mostly consisting of infections. A total of 20 (19.6%) patients reported zevor-cel related infections and infestations, which were mostly grade 3, pneumonia being the most common infection diagnosed in 16 patients (15.7%). Other common zevor-cel related SAEs included platelet count decreased in 15 (14.7%) and CRS in 3 patients (2.9%).

Deaths were reported in 20 patients (19.6%), with 13 deaths due to progressive disease, 4 due to AEs, 1 patient due to progressive disease along with AE, 1 due to septic shock and 1 due to covid-19 pneumonia (not reported as AE due to AE collection rules). Only 1 (1.0%) was determined to be related to zevor-cel, which was caused by pneumonia, that occurred 149 days post infusion.

Four cases of second primary malignancies were reported on the study, one each of basosquamous carcinoma of skin, acute myeloid leukaemia, tumour of ampulla of Vater, and squamous cell carcinoma of the cervix (with HPV16 positive), all were deemed unrelated to zevor-cel. No zevor-cel related autoimmune diseases were reported. All patients tested negative for replication competent lentivirus (RCL) in the study.

#### Pharmacokinetics, immunogenicity and exposure-response relationship

**Fig. 5:**
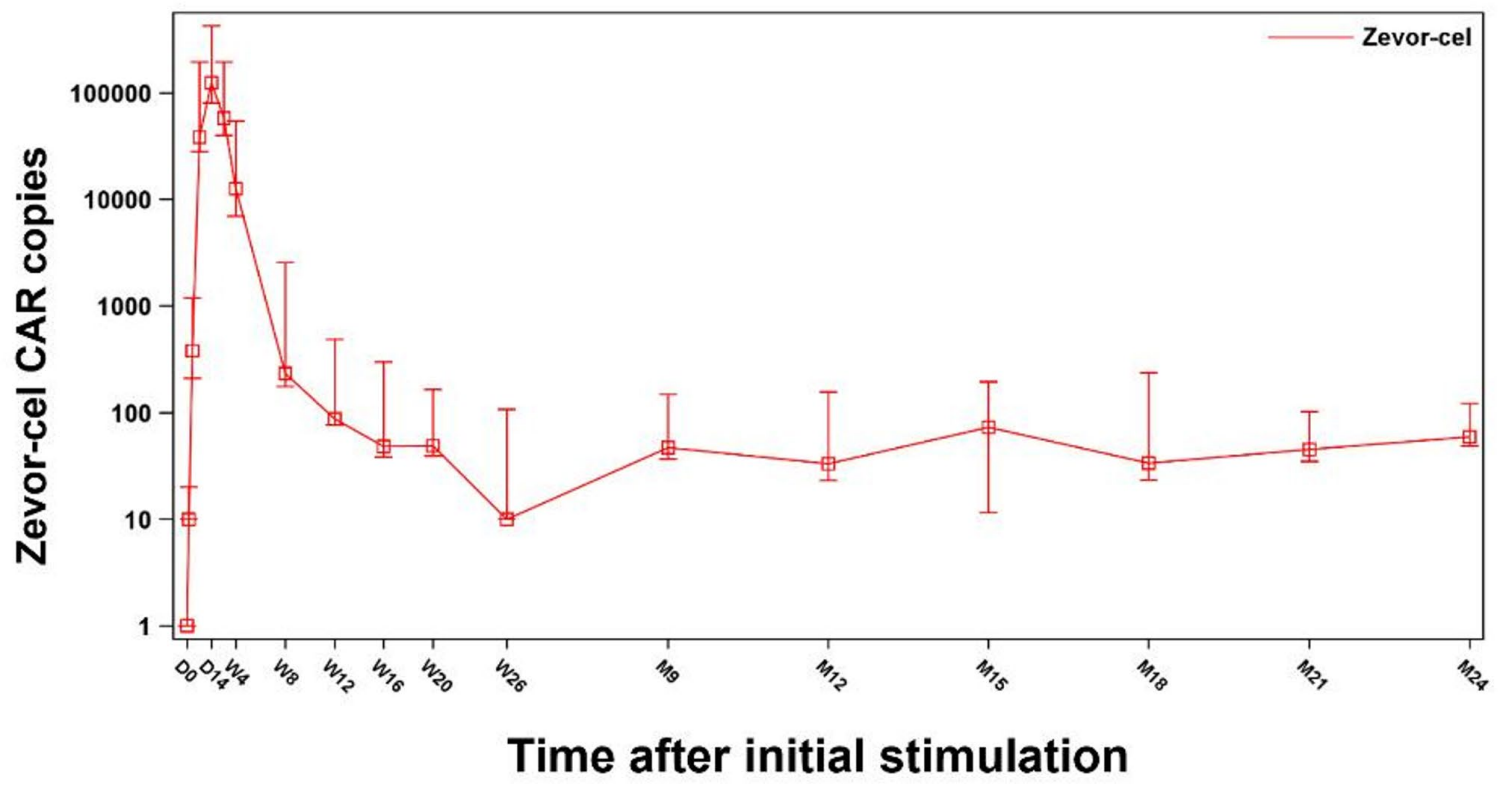
Copies of zevor-cel as detected by qPCR with time. Peripheral blood zevor-cel expansion was measured as copies per microgram of genomic DNA by qPCR. *D* day; *CAR* chimeric antigen receptor; *M* month; *qPCR* quantitative PCR; *W* week

All 102 patients who received zevor-cel infusion were included in the PK analysis. Quantifiable levels of zevor-cel were detected in 93 (91.2%) patients within 3 days following infusion. The median C_max_ was 202543.5 (range: 9544, 1128461) copies/ µg gDNA and the median T_max_ was 14.0 (range: 7, 22) days. The median T_last_ was 140.0 (range: 26–740) days. The median area under the curve (AUC) AUC_0 − t_ and AUC_0 − inf_ values of transgene copies were 2193780.7 (range: 59293.9, 13937955.4) and 2448492.6 (range: 174872.7, 13941661.8) day*copies/µg gDNA, respectively. Median AUC_0 − D7_ to AUC_0 − W26_ of the transgene copies were steadily increased from 77228.0 (range: 478.9, 1157968.0) day*copies/µg gDNA to 2232862.9 (range: 177306.8, 13922819.3) day*copies/µg gDNA (Fig. 5).

Anti-drug antibodies (ADAs) were detected in 21 patients (20.6%) and neutralizing antibodies (NABs) were detected in 16 patients (15.7%) until the data cut-off date.

The AUC_0 − 28_ values were observed to be elevated in patients with CRS in comparison to those without CRS; however, this difference did not reach statistical significance (median values of 2008586.8 vs. 1559700.1 copies/µg gDNA; *P* = 0.2557) (see Supplementary Fig. 1). In contrast, the C_max_ was significantly greater in patients with treated CRS than in those with untreated CRS (median values of 24155 vs. 76383.0 copies/µg gDNA; *P* = 0.0095). Furthermore, C_max_ was also higher in patients with treated CRS compared to those without CRS, although this difference was not statistically significant (median values of 214923.5 vs. 133426.5 copies/µg gDNA; *P* = 0.3137) (see Supplementary Fig. 2). Among the cohort, 92 patients with CRS exhibited a response rate of 95.7%, while 10 patients without CRS had a response rate of 60.0%, with this difference being statistically significant (*P* = 0.0028) (see Supplementary Fig. 3). Additionally, 90 patients with CRS and 9 patients without CRS demonstrated MRD-negative (10^− 5^) rates of 94.4% and 66.7%, respectively, with this difference also being statistically significant (*P* = 0.0235) (see Supplementary Fig. 4). The concentrations of cytokines IL-2, IL-6, IL-10, and IFN-γ were found to be significantly higher in patients with CRS compared to those without CRS (all P values < 0.05) (see Supplementary Fig. 5).

**Table 1:** Baseline characteristics and prior treatments

Characteristics	Patients (*N* = 102)
Age, median (range), years	59.5 (38–75)
18 – <65	71 (69.6)
65–75	31 (30.4)
>75	0
Sex, n (%)	
Male	55 (53.9)
Female	47 (46.1)
Time since diagnosis, median (range), years	3.6 (0.7–16)
Lines of previous anti-MM treatment, median (range)	4.0 (3–15)
Type of immunoglobulin, n (%)	102 (100%)
IgG	58 (56.9)
IgA	20 (19.6)
IgM	0
IgD	4 (3.9)
IgE	1 (1.0)
Light Chain	19 (18.7)
ECOG PS, n (%)	
0	54 (52.9)
1	48 (47.1)
>1	0
ISS Stage, n (%)	102 (100)
I	24 (23.5)
II	39 (38.2)
III	39 (38.2)
BCMA expression rate, n (%)^b^	89
<50%	16 (15.7)
≥50%	73 (71.6)
Plasma cells in bone marrow, n (%)	
<50%	83 (81.4)
≥50%	17 (16.7)
Disease risk classification, n (%)	
High risk^a^	46 (45.1)
Not detected	56 (54.9)
Cytogenetic Features, (%)	
Del (17p13.1)	
(–)	67 (65.7)
(+)	22 (21.6)
t (4;14)	
(–)	59 (57.8)
(+)	29 (28.4)
t (14;16)	
(–)	86 (84.3)
(+)	2 (2.0)
t (14;20)	
(–)	87 (85.3)
(+)	1 (1.0)
Presence of extramedullary disease, n (%)^c^	11 (10.8)
Proteasome inhibitors, n (%)	102 (100)
Bortezomib	101 (99.0)
Carfilzomib	102 (100)
Ixazomib	101 (99.0)
IMiDs, n (%)	102 (100)
Lenalidomide	101 (99.0)
Pomalidomide	34 (33.3)
Thalidomide	55 (53.9)
Anti-CD38 monoclonal antibody, n (%)	30 (29.4)
Daratumumab	21 (20.6)
Other anti-CD38 monoclonal antibodies	9 (8.8)
Prior autologous stem-cell transplantation	24 (23.5)
Double-class exposed, n(%)^d^	102 (100)
Triple-class exposed, n(%)^e^	30 (29.4)
Penta-class exposed, n(%)^f^	6 (5.9)
Double-class refractory, n (%)^g^	91 (89.2)
Triple-class refractory, n (%)^h^	23 (22.5)
Bridging therapy, n (%)	26 (25.5)

Baseline is defined as the last non-missing values taken prior to lymphodepletion chemotherapy

*BCMA* B-cell maturation antigen; *CD38* cluster of differentiation 38; *ECOG PS* Eastern Cooperative Oncology Group Performance Status Scale; *IMiD* immunomodulatory drug; *ISS* International Staging System; *MM* multiple myeloma

^a^High risk was defined as the presence of one or more high risk cytogenetics: del (17p), t (4;14), t (14;16), and t (14;20). These were assessed using fluorescence in situ hybridization (FISH) in bone marrow, whereas the rest are defined as ' not detected ‘

^b^BCMA expression rate is detected in abnormal plasma cell membrane of bone marrow

^c^Extramedullary Disease is defined as the presence at least one measurable soft tissue or paramedullary plasmacytomas as assessed by the MRI/CT or CT component of PET/CT

^d^Double-class exposed is defined as exposed to at least one protease inhibitor (such as bortezomib, carfilzomib or ixazomib) and at least one IMiD (including lenalidomide, pomalidomide or thalidomide)

^e^Triple-class exposed is defined as exposed to at least one protease inhibitor (such as bortezomib, carfilzomib or ixazomib) and at least one IMiD (including lenalidomide, pomalidomide or thalidomide) and at least 1 anti-CD38 antibody (such as daratumumab or other anti-CD38 monoclonal antibodies or antibody drug conjugates)

^f^Penta-drug exposed is defined as exposed to at least two protease inhibitor (such as bortezomib, carfilzomib or ixazomib) and at least two IMiD (including lenalidomide, pomalidomide or thalidomide) and at least 1 anti-CD38 antibody (such as daratumumab or other anti-CD38 monoclonal antibodies or antibody drug conjugates)

^g^Double-class refractory is defined as refractory to at least one protease inhibitor (such as bortezomib, carfilzomib, ixazomib) and at least one IMiD (including lenalidomide, pomalidomide, thalidomide) and the reason for discontinuation is disease progression, lack of efficacy or other

^h^Triple-class refractory is defined as refractory to at least one protease inhibitor (such as bortezomib, carfilzomib or ixazomib) and at least one IMiD (such as lenalidomide, pomalidomide or thalidomide) and at least 1 anti-CD38 targeting therapies (such as daratumumab or other anti-CD38 monoclonal antibodies or antibody drug conjugates) and the reason for discontinuation is disease progression, lack of efficacy or other

**Table 2:** Summary of responses to zevor-cel

	Zevor-cel 1.5 × 10^8^ (*N* = 102)
Best Overall Response, n (%)	
Stringent Complete Response (sCR)	70 (68.6)
Complete Response (CR)	3 (2.9)
Very Good Partial Response (VGPR)	20 (19.6)
Partial Response (PR)	1 (1.0)
Minimal Response (MR)	1 (1.0)
Stable Disease (SD)	2 (2.0)
Progressive Disease (PD)	4 (3.9)
Unknown (UNK)	1 (1.0)
Objective Response Rate (ORR), n (%)	94 (92.2)
95% CI	(85.13, 96.55)
CR/sCR rate, n (%)	73 (71.6)
95% CI	(61.78, 80.06)
sCR rate, n (%)	70 (68.6)
95% CI	(58.69, 77.45)
CR rate, n (%)	3 (2.9)
95% CI	(0.61, 8.36)
VGPR or better rate, n (%)	93 (91.2)
95% CI	(83.91, 95.89)
MRD negativity within patients with MRD results (< 10^− 5^), n (%)^a^	91 (91.9)
95% CI	(84.70, 96.45)
CR/sCR patients with MRD assessment, n	73
MRD negativity within CR/sCR patients with MRD results (< 10^− 5^), n (%)^b^	73 (100)
95% CI	(95.07, 100.00)
VGPR or better patients with MRD assessment, n	92
MRD negativity within VGPR or better patients with MRD results (< 10^− 5^), n (%)^c^	88 (95.7)
95% CI	(89.24, 98.80)

As of cut-off date, the 102 patients infused with zevor-cel have completed a median of 20.3 months follow-up, and 32 patients who entered the long-term follow-up have been followed for at least 23.3 months after receiving zevor-cel infusion

95% confidence interval was calculated using the Clopper-Pearson method

*ORR* objective response rate; *sCR* stringent complete response; *CR* complete response; *VGPR* very good partial response; *PR* partial response; *MR* minimal residual disease; *SD* stable disease; *PD* progressive disease; *UNK* unevaluable

^a^Percentage calculation is based on patients tested by MRD as the denominator

^b^Percentage calculation is based on patients confirmed with CR and above (IRC assessment) and tested by MRD as the denominator

^c^Percentage calculation is based on patients confirmed with VGPR and above (IRC assessment) and tested by MRD as the denominator

**Table 3:** Summary of zevor-cel-related adverse event by SOC, PT and grade (all grade ≥ 20%)

	Zevor-cel 1.5 × 10^8^ (*N* = 102)	
SOC, *n*(%) PT, *n* (%)	≥ Grade 3	Total
Number of patients with at least one zevor-cel -related AE	98 (96.1)	102 (100)
Investigations	95 (93.1)	101 (99.0)
Neutrophil count decreased	91 (89.2)	95 (93.1)
White blood cell count decreased	78 (76.5)	93 (91.2)
Lymphocyte count decreased	71 (69.6)	86 (84.3)
Platelet count decreased	61 (59.8)	86 (84.3)
Serum ferritin increased	16 (15.7)	70 (68.6)
C-reactive protein increased	7 (6.9)	65 (63.7)
Aspartate aminotransferase increased	22 (21.6)	57 (55.9)
Blood lactate dehydrogenase increased	5 (4.9)	57 (55.9)
Fibrin D dimer increased	8 (7.8)	48 (47.1)
Alanine aminotransferase increased	4 (3.9)	37 (36.3)
Blood alkaline phosphatase increased	4 (3.9)	27 (26.5)
Blood creatinine increased	3 (2.9)	26 (25.5)
Lymphocyte count increased	1 (1.0)	26 (25.5)
Fibrin degradation products increased	4 (3.9)	25 (24.5)
Gamma-glutamyltransferase increased	6 (5.9)	25 (24.5)
Blood fibrinogen decreased	2 (2.0)	23 (22.5)
Alpha hydroxybutyrate dehydrogenase increased	3 (2.9)	22 (21.6)
Procalcitonin increased	1 (1.0)	21 (20.6)
Immune system disorders	10 (9.8)	98 (96.1)
Cytokine release syndrome	7 (6.9)	92 (90.2)
Hypogammaglobulinemia	5 (4.9)	27 (26.5)
General disorders and administration site conditions	13 (12.7)	96 (94.1)
Pyrexia	11 (10.8)	95 (93.1)
Blood and lymphatic system disorders	36 (35.3)	70 (68.6)
Anaemia	35 (34.3)	67 (65.7)
Metabolism and nutrition disorders	23 (22.5)	65 (63.7)
Hypokalaemia	14 (13.7)	41 (40.2)
Hypocalcaemia	9 (8.8)	39 (38.2)
Hyponatraemia	1 (1.0)	27 (26.5)
Hypoalbuminemia	0	23 (22.5)
Hyperuricaemia	1 (1.0)	21 (20.6)
Hypophosphatemia	0	21 (20.6)
Infections and infestations	28 (27.5)	54 (52.9)
Pneumonia	20 (19.6)	24 (23.5)
Gastrointestinal disorders	1 (1.0)	41 (40.2)
Diarrhoea	0	21 (20.6)
Vascular disorders	6 (5.9)	27 (26.5)
Hypotension	5 (4.9)	24 (23.5)

MedDRA (MedDRA 24.1) was used for coding

*PT* preferred term; *SOC* system organ class; *TEAE* treatment-emergent adverse event

**Table 4:** Summary of CRS and ICANS

CAR T-cell associated AEs *n* (%)	Zevor-cel 1.5 × 10^8^ (*N* = 102)
CRS*	
Any grade, n (%)	92 (90.2)
1	52 (56.5)
2	33 (35.9)
3	5 (5.4)
4	2 (2.2)
5	0
Time to first onset, median, d (range)	4 (1–14)
Duration, median, d (range)	6 (1–22)
Tocilizumab use, n (%)	66 (71.7)
Corticosteroid use, n (%)	25 (27.2)
ICANS*	
Any grade, n (%)	2 (2.0)
1	2 (100)
2	0
≥ 3	0
Time to first onset, median, d (range)	15.5 (12–19)
Duration, median, d (range)	7 (3–11)
Tocilizumab use, n (%)	0
Corticosteroid use, n (%)	0
Patients with either CRS or ICANS	92 (90.2)

The percentages by maximum grade are based on the number of patients with CRS/ICANS

Time to onset: Onset date of the first CRS/ICANS–zevor-cel infusion start date + 1

Duration: Last CRS/ICANS end date–Earliest CRS/ICANS onset date + 1, including the intervening non-event days if a patient reports multiple CRS/ICANS

*AE* adverse event; *CRS* cytokine release syndrome; *d* day; *ICANS* immune effector cell-associated neurotoxicity syndrome

*Graded according to ASTCT Consensus Grading for Cytokine Release Syndrome and Neurologic Toxicity Associated with Immune Effector Cells

## Discussion

While CAR T-cell therapies have improved outcomes in RRMM in many parts of the world, their benefit to Chinese patients has been limited due to accessibility, healthcare infrastructure disparities, and socioeconomic barriers. Zevor-cel could serve as an efficacious therapeutic option for patients with RRMM as evidenced by the ORR of 92.2% coupled with an CR/sCR rate of 71.6% observed in our study. In the pivotal phase 2 KarMMa trial [12], ide-cel reported an ORR of 72% with a sCR rate of 28% whereas in the phase 1b/2 CARTITUDE-1 study cilta-cel achieved an ORR of 97.9% with a sCR rate of 80.4% [14]. Meanwhile, the LEGEND-2 study conducted in China with cilta-cel reported an ORR of 87.8% with CR rate of 73% [17]. In the phase 1b/2 FUMANBA-1 trial conducted in China, eque-cel reported an ORR of 96.0%, with 74.3% of the patients with a CR or better [25]. Durability of response (DOR) observed with BCMA-targeting CAR-T therapies has been variable; in KarMMa study ide-cel achieved a median DOR of 11 months whereas cilta-cel achieved a median DOR of 33.9 months in the CARTITUDE − 1 study (median follow-up of 33.4 months), however, in the LEGEND-2 study conducted in China, cilta-cel reported a median DOR of 23 months (median follow-up of 65.4 months). Eque-cel reported a median DOR of 12.3 months in China with a median follow-up of 13.8 (0.4–27.2) months in the phase 1b/2 FUMANBA-1 trial [25]. The median DOR of zevor-cel data were not mature at median follow-up of 20.3 months with 62.4% (95% CI: 51.2%–71.6%) patients remaining event-free at 18 months. Overall, the response rates and the DOR observed with zevor-cel in our study appear to be numerically superior to those observed with ide-cel and broadly comparable to those observed with studies conducted with cilta-cel and eque-cel in China. However, any such indirect comparisons and the interpretations should be approached with caution. Notably, the OS rates of zevor-cel at 12, 18 and 30 months were 90.2%, 83.3% and 79.4% respectively, which are quite high and reflect zevor-cel’s advantage in long-term survival.

The analyses for subgroups with sample size greater than 20% of the 102 study population who received zevor-cel infusion of the pivotal Phase II stage of LUMMICAR STUDY 1, including age (< 65 years versus ≥ 5 years), International Staging System (ISS; I/II versus III), and high-risk cytogenetics, indicated that the clinical efficacy of zevor cel is not significantly impacted by baseline characteristics suggesting that even patients with RRMM with poor prognostic factors may benefit from zevor-cel [26]. 

Despite the compelling efficacy reported with BCMA-targeting cell therapies, safety concerns continue to linger. Overall safety profile of zevor-cel in the LUMMICAR-1 study has been reassuring; CRS of any grade was reported in 92 (90.2%) patients which were predominantly grade1/2 (*n* = 85; 83.3%) with grade 3 or 4 events occurring in 7 (6.9%) patients, all of whom fully recovered. This is overall comparable to the observations across CARTITUDE-1 and 4 studies of cilta-cel (overall CRS incidence of 84% with 4% patients experiencing Grade 3 or higher events) [27] and KarMMa and KarMMa-3 studies of ide-cel (overall CRS incidence of 89% with 7.9% patients suffering from Grade 3 or higher events including 1 death) [28]. 

Neurological toxicities associated with CAR-T cell therapies can be debilitating. Among patients receiving cilta-cel in the CARTITUDE-1 and CARTITUDE-4 studies, one or more neurologic toxicities occurred in 24% (69/285), including ≥ grade 3 events in 7% (19/ 285) of patients. Subtypes of neurologic toxicities included ICANS in 13%, peripheral neuropathy in 7%, cranial nerve palsies in 7% and parkinsonism in 3%. In patients receiving ide-cel in the KarMMa and KarMMa-3 studies, neurotoxicity attributable to ide-cel occurred in 40% (139/349), including Grade 3 or 4 events in 4.6% (16/349) of the patients. The most frequent CAR T cell-associated manifestations included encephalopathy including ICANS (21%), headache (15%), dizziness (8%), delirium (6%), and tremor (6%). In contrast, we report zevor-cel-related nervous system disorders in 18.6% of patients in our study. The most common zevor-cel-related nervous system disorders were dizziness (7.8%) and headaches (7.8%), both of which were at grade 1 or 2. There were no incidence of zevor-cel related grade 3 or higher neurotoxicity; only 2 patients experienced grade 1 ICANS.

The potential for oncogenesis caused by genomic integration or by other mechanisms is an ongoing concern with the use of CAR T-products. A recent report by the US FDA highlighted 22 cases of T-cell malignancies that occurred after treatment with CAR T-products [29]. Among patients receiving cilta-cel in the CARTITUDE-1 and CARTITUDE-4 studies, myeloid neoplasms occurred in 5% (13/285) of patients (9 cases of myelodysplastic syndrome, 3 cases of acute myeloid leukaemia, and 1 case of myelodysplastic syndrome followed by acute myeloid leukaemia) [11]. In our study, four second primary malignancies were reported (1 case of basosquamous carcinoma of skin, 1 case of acute myeloid leukaemia, 1 case of tumour of ampulla of Vater, 1 case of squamous cell carcinoma of the cervix. It is possible that the difference in the incidence of new malignancies could be due to the difference in follow-up durations, however, any potential safety advantages of zevor-cel may not be ruled out.

In our study, only one death was determined to be related to zevor-cel, which was caused by pneumonia that occurred 149 days post infusion. Most AEs were classified as grade 1 or 2, allowing for outpatient management in many instances. In a phase 2 study of cilta-cel with a median follow-up of 26.4 months, all 48 patients who had received cilta-cel experienced ≥ 1 grade 3/4 TEAEs and a total of 12 deaths post cilta-cel infusion (8 considered treatment-related) were reported [30]. In a phase 1b/2 study of CT103A with median follow-up of 147 days, ≥ grade 3 treatment-related AEs were reported with the most common ≥ grade 3 treatment-related AEs being hematologic [31]. Li et al. reported that although CAR T-cell therapy is effective in treating R/R MM, severe hematological toxicity (HT) remains an intractable issue, and prolonged HT (PHT) on Day 28 post-infusion was associated with poor PFS and OS in MM patients after CAR T-cell therapy [32]. In our study, the majority of grade ≥ 3 haematological toxicities resolved within 30 days post infusion, which further supports its favorable safety profile and potential better OS benefit. Taken together, zevor-cel appears to confer a potentially superior safety advantage over other approved BCMA-targeting CAR-T cell therapies with the caveats of cross-trial comparisons. It is plausible that fully human construct of zevor-cel confers some safety advantages. The relatively lower doses of fludarabine (25 mg/m^2^) and cyclophosphamide (300 mg/m^2^) for 3 days that utilized in our study for lymphodepletion may also have contributed to the favourable safety observations. For heavily pre-treated patients with RRMM who often suffer from numerous comorbidities, frailty, and limited bone marrow reserve, zevor-cel could serve as an optimal therapeutic choice due to its distinct safety profile.

### Limitations of this study

The sample size of this study was relatively small and there was heterogeneity in disease type and duration. There was no control arm in the study to compare the relative efficacy of zevor-cel to other available standard-of-care treatment options. Currently, a larger multi-center study named LUMMICAR STUDY 2 is underway in the USA and Canada (NCT03915184) alongside a real-world study (NCT06659770) in China to further investigate the long-term safety and efficacy of zevor-cel in patients with RRMM.

## Conclusion

In heavily pre-treated patients with RRMM, zevor-cel demonstrates robust anti-myeloma activity and induces deep and durable clinical responses with a manageable safety profile.

## Supplementary Information

Below is the link to the electronic supplementary material.

Supplementary Material 1

## Data Availability

No datasets were generated or analysed during the current study.
