# Supplementary material for: Phase II study of zevorcabtagene autoleucel, a fully human BCMA-targeting CAR T cell therapy, in patients with relapsed/refractory multiple myeloma
Source: Exp Hematol Oncol. 2025 Sep 30;14:119. doi: 10.1186/s40164-025-00710-y (PMC12482220; doi:10.1186/s40164-025-00710-y)
Supplement: Supplementary file 1 — Supplementary Material 1 [file 40164_2025_710_MOESM1_ESM.docx]

**SUPPLEMENTARY INFORMATION**

**PHASE II STUDY OF FULLY HUMAN BCMA-TARGETING CAR-T CELLS (ZEVORCABTAGENE AUTOLEUCEL) IN PATIENTS WITH RELAPSED/ REFRACTORY MULTIPLE MYELOMA**

**Authors**

Wenming Chen, MD*^1^, Chengcheng Fu, MD*^2^, Baijun Fang, MD ^3^, Aibin Liang, MD ^4^, Zhong-Jun Xia, MSc^5^, Yanjuan He, MD ^6^, Jin Lu, MD ^7^, Hui Liu, MD ^8^, Ming Hou, MD ^9^, Zhen Cai, MD ^10^, Wei Yang, MD ^11^, Siguo Hao, MD ^12^, Songfu Jiang, MD ^13^, Hongmei Jing, MD ^14^, Jing Liu, MD ^15^, Xin Du, MD ^16^, Rong Fu, MD ^17^, Heng Mei, MD ^18^, Zunmin Zhu, MD ^19^, Yanli Yang, MSc^20^, Hong Liu, MD ^21^, Xingxing Meng^22^, Nishanthan Rajakumaraswamy^22^, Daijing Yuan^22^, Huamao Wang^22^, Zonghai Li^22^

**Affiliations**

^1^Beijing Chao-Yang Hospital, Capital Medical University, Department of Hematology, Beijing, China,

^2^The First Affiliated Hospital of Soochow University, Suzhou, China,

^3^Henan Cancer Hospital, Zhengzhou, China,

^4^Tongji Hospital of Tongji University, Shanghai, China,

^5^Sun Yat-sen University Cancer Center, Department of Hematology, Guangzhou, China,

^6^Xiangya Hospital, Central South University, Department of Hematology, Changsha, China,

^7^Peking University People's Hospital, Beijing, China,

^8^Beijing Hospital, Department of Hematology, Beijing, China,

^9^Qilu Hospital of Shandong University, Department of Hematology, Jinan, China,

^10^The First Affiliated Hospital, Zhejiang University School of Medicine, Hangzhou, China,

^11^Shengjing Hospital of China Medical University, Shenyang, China,

^12^Xinhua Hospital Affiliated to Shanghai Jiaotong University School of Medicine, Shanghai, China,

^13^The First Affiliated Hospital of Wenzhou Medical University, Wenzhou, China,

^14^Peking University Third Hospital, Department of Hematology, Beijing, China,

^15^The Third Xiangya Hospital of Central South University, Department of Hematology, Changsha, China,

^16^The Second People's Hospital of Shenzhen, The First Affiliated Hospital of Shenzhen University, Division of Hematology, Shenzhen, China,

^17^Tianjin Medical University General Hospital, Department of Hematology, Tianjin, China,

^18^Union Hospital, Tongji Medical College, Huazhong University of Science and Technology, Wuhan, China,

^19^Henan Provincial People's Hospital, Department of Hematology, Zhengzhou, China,

^20^The First Affiliated Hospital of Bengbu Medical College, Bengbu, China,

^21^Affiliated Hospital of Nantong University, Nantong, China,

^22^CARsgen Therapeutics Co. Ltd, Shanghai, China

***Corresponding author （These authors contributed equally to this work and should be considered co-first authors.）**


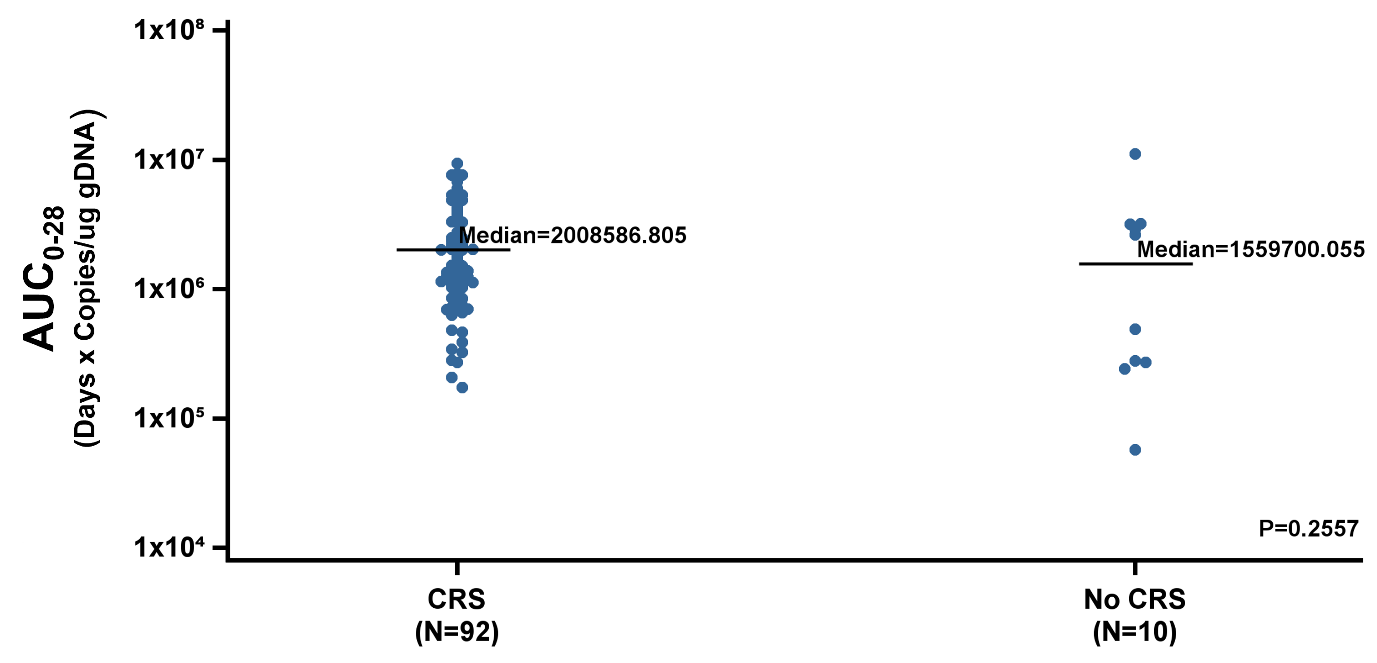


AUC_0-28_ was defined as cumulative levels of CAR transgene in patients’ peripheral blood over the first 28 days. The data analysis was conducted utilizing Pharmacokinetic Analysis Set.

Note: Each point represents data from one patient and the black line indicates the median. The two-sided P value was calculated using Kruskal-Wallis test.

Abbreviations: AUC, area under the curve; CAR, chimeric antigen receptor; CRS, cytokine release syndrome; gDNA, genomic deoxyribonucleic acid.

**Supplementary Figure 1.** AUC_0–28_ of zevor-cel based on the occurrence of CRS (Pharmacokinetic Analysis Set)


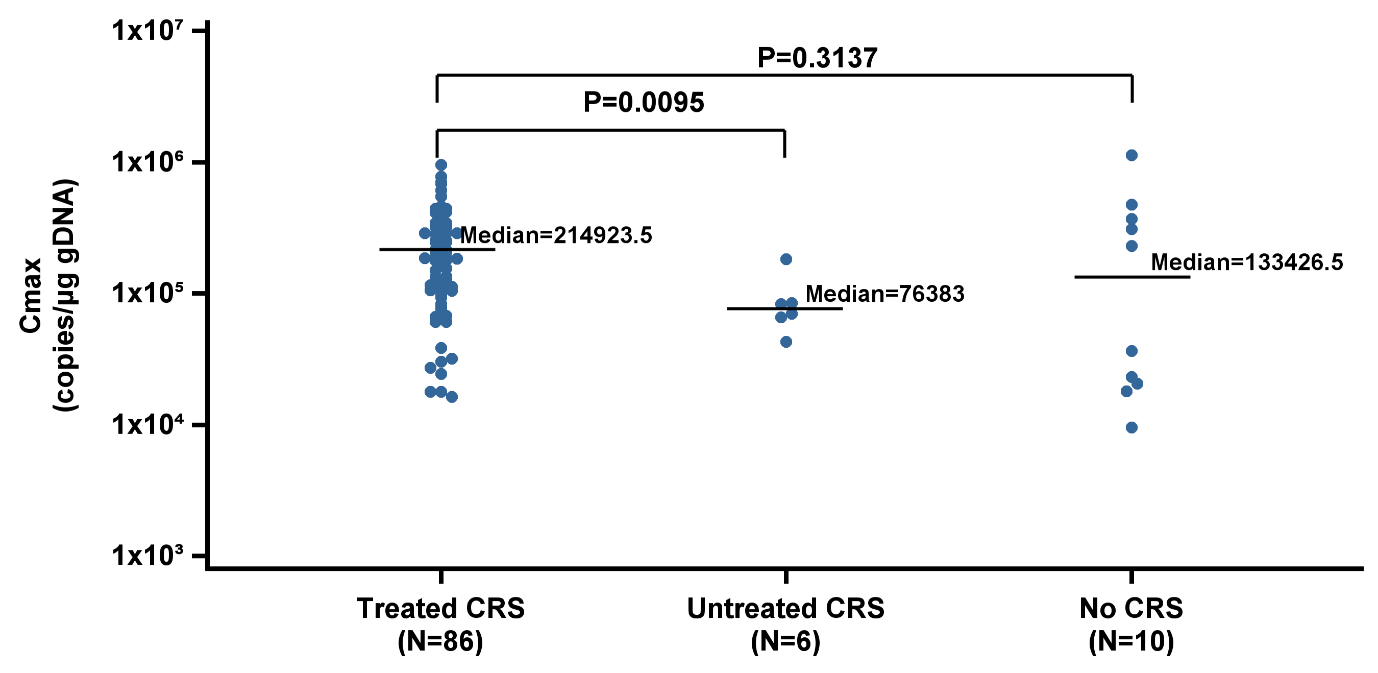


Note: Each point represents data from one patient and the black line indicates the median. Two-sided P values were calculated using the Kruskal-Wallis test.

Abbreviations: C_max_, peak serum concentration; CRS, cytokine release syndrome; gDNA, genomic deoxyribonucleic acid.

**Supplementary Figure 2**. C_max_ in patients with CRS resolved with or without treatment and in those without. (Pharmacokinetics concentration set)


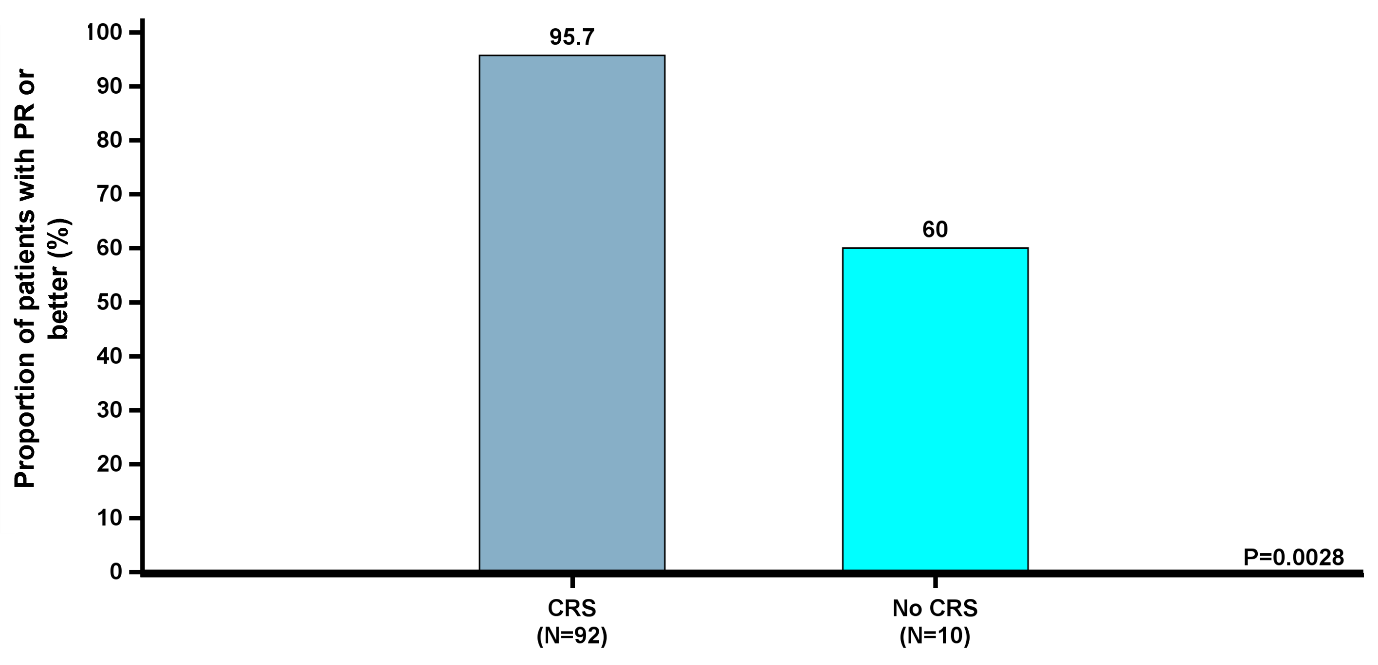


Note：p value is derived using Fisher's exact test.

Abbreviations: CRS, cytokine release syndrome; PR, partial response.

**Supplementary Figure 3**. Objective response rate by occurrence of CRS (Efficacy evaluation set)


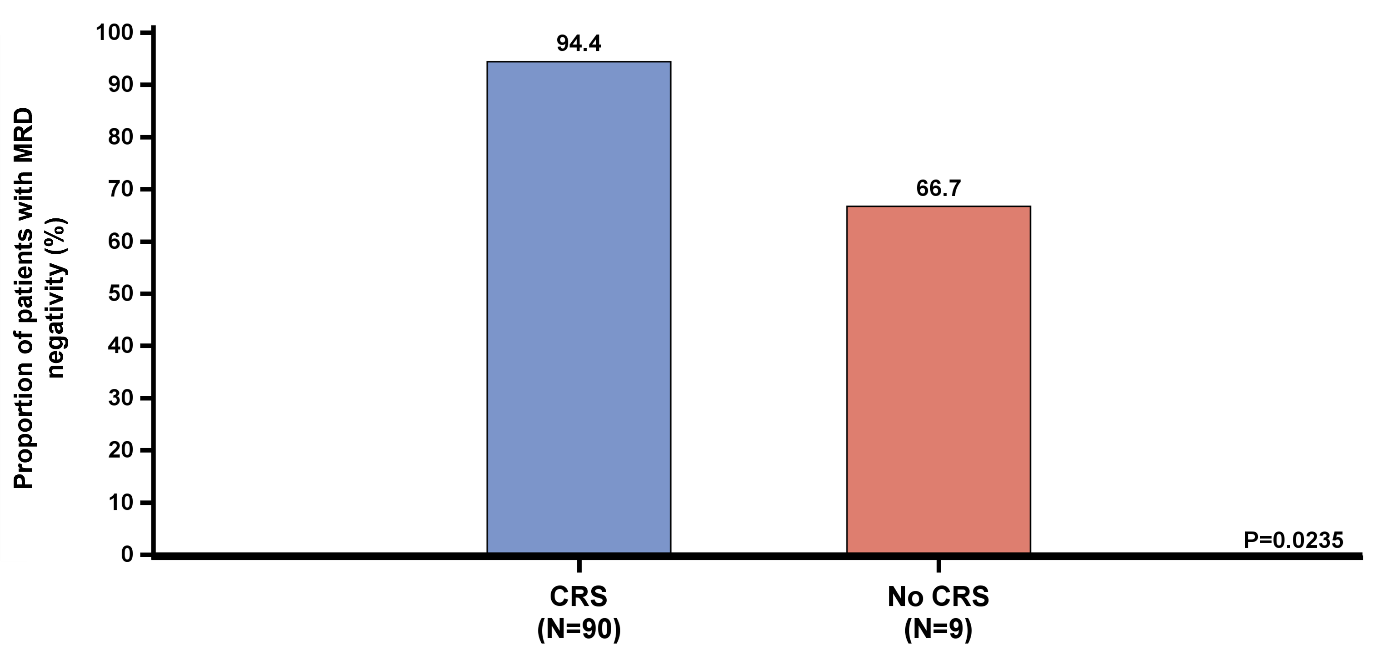


P values were derived using Fisher's exact test.

Note：Patients with at least one MRD test result are included.

Abbreviations: CRS, cytokine release syndrome; MRD, minimal residual disease.

**Supplementary Figure 4**. MRD negativity (10^-5^) by occurrence of CRS


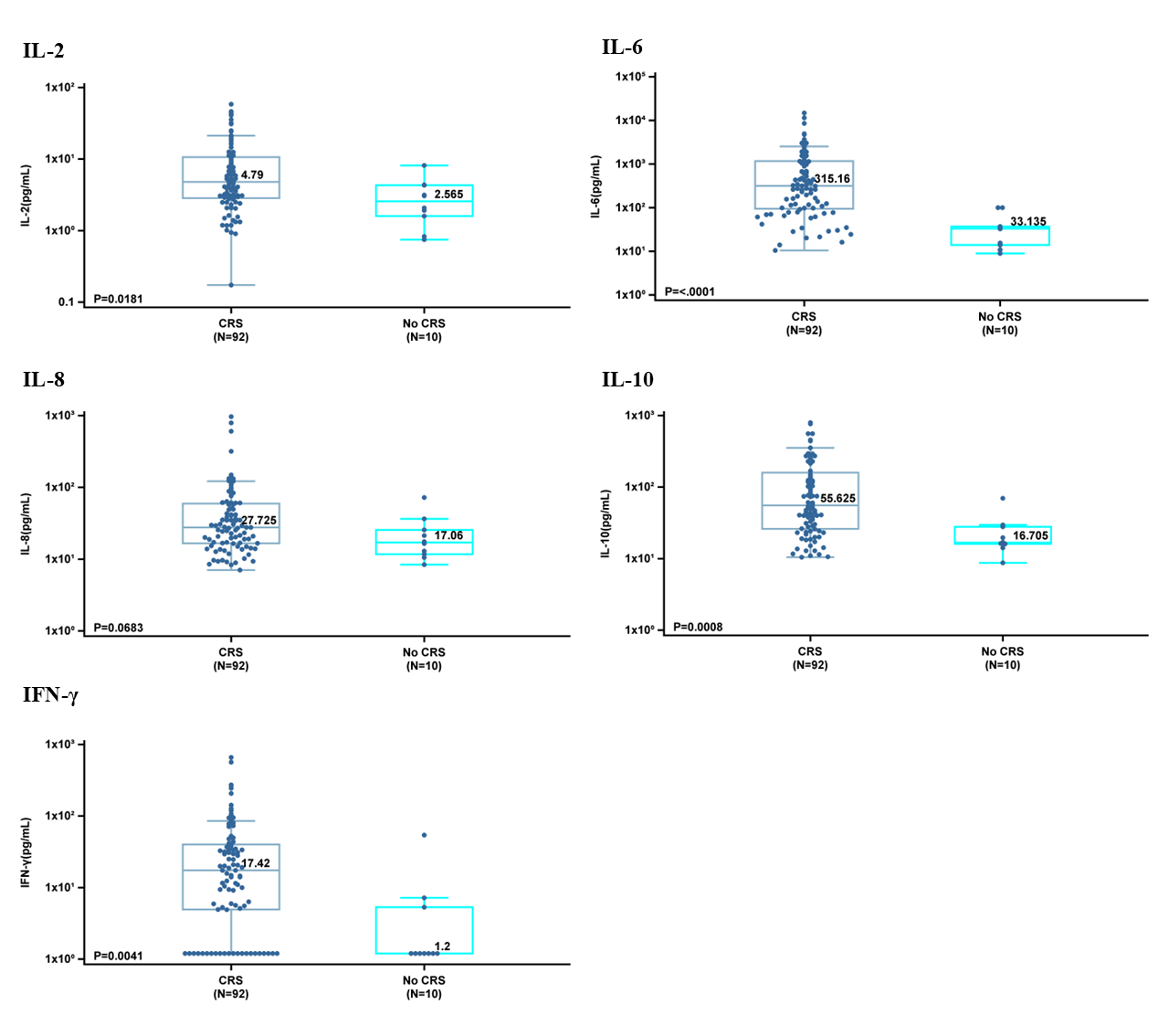


Abbreviations: IL, interleukin; IFN, interferon.

**Supplementary Figure 5**. **Median cytokine concentrations over time (Full Analysis Set)**
